# Potential genetic robustness of *Prnp* and *Sprn* double knockout mouse embryos towards ShRNA-lentiviral inoculation

**DOI:** 10.1186/s13567-022-01075-4

**Published:** 2022-07-07

**Authors:** Andrea Rau, Bruno Passet, Johan Castille, Nathalie Daniel-Carlier, Alexandre Asset, Jérome Lecardonnel, Marco Moroldo, Florence Jaffrézic, Denis Laloë, Katayoun Moazami-Goudarzi, Jean-Luc Vilotte

**Affiliations:** 1 Université Paris-Saclay, INRAE, AgroParisTech, GABI, 78350 Jouy-en-Josas, France; 2 BioEcoAgro Joint Research Unit, INRAE, Université de Liège, Université de Lille, Université de Picardie Jules Verne, 80203 Péronne, France

**Keywords:** Mouse, prion, Shadoo, lentivirus, ShRNA, robustness

## Abstract

The Shadoo and PrP prion protein family members are thought to be functionally related, but previous knockdown/knockout experiments in early mouse embryogenesis have provided seemingly contradictory results. In particular, Shadoo was found to be indispensable in the absence of PrP in knockdown analyses, but a double-knockout of the two had little phenotypic impact. We investigated this apparent discrepancy by comparing transcriptomes of WT, *Prnp*^*0/0*^ and *Prnp*^*0/0*^*Sprn*^*0/0*^ E6.5 mouse embryos following inoculation by *Sprn*- or *Prnp*-ShRNA lentiviral vectors. Our results suggest the possibility of genetic adaptation in *Prnp*^*0/0*^*Sprn*^*0/0*^ mice, thus providing a potential explanation for their previously observed resilience.

## Introduction

The prion protein PrP, encoded by *Prnp*, is strongly associated with several neurodegenerative diseases; in particular, misfolded isoforms of PrP are thought to be a key component of the infectious prions that cause Transmissible Spongiform Encephalopathy. PrP is evolutionarily related to another member of the prion protein family, Shadoo, which is encoded by *Sprn* [1]. However, their individual biological functions and the complex interrelationship between the two remain poorly characterized. Previous single and double knockdown experiments in early mouse embryogenesis have provided seemingly contradictory results. Individual genetic invalidations yielded little phenotypic impact beyond resistance to prion infection for *Prnp*-knockout mice. Knockdown of *Sprn* in *Prnp*-knockout embryos was found to induce early embryonic lethality as early as E7.5 linked to the developmental failure of the trophectoderm-derived compartment [2]. Although these results together appeared to suggest a potential biological redundancy of the two proteins, double genetic invalidations of *Prnp* and *Sprn* in mice with various genetic backgrounds [3, 4] did not confirm this hypothesis; we note that all experiments involved FVB/N genetic backgrounds, obtained either by introgression following embryonic stem (ES) cell manipulations or direct use of nucleases. These apparently contradictory observations could result from a genetic compensation in invalidated animals [5] or from an increased robustness [6].

In the present report, we comparatively assessed, at the transcriptomic level, the impact of *Prnp* and *Sprn* knockout in E6.5 mouse embryos and its consequences following inoculation with *ShRNA*-lentiviral vectors at the one cell stage.

### Transgenic lines and lentiviral inoculations

**Figure 1:**
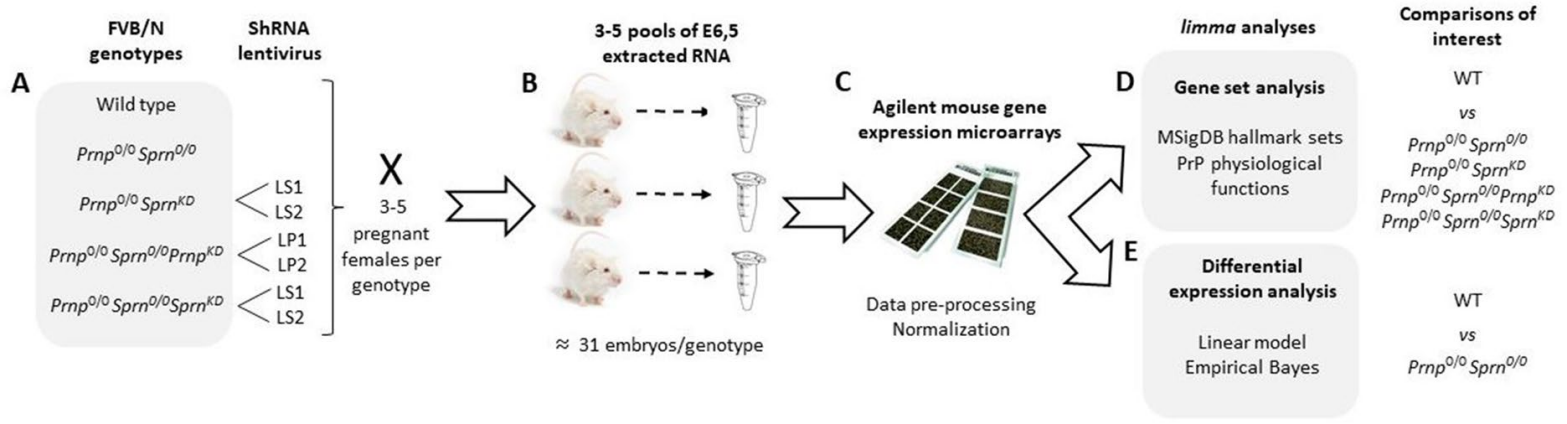
**Schematic representation of study design and analysis.** **A** Transgenic lines and lentiviral inoculations. **B** Embryo collection. **C** Transcriptomic analyses. **D** Hallmark gene set analysis; and **E** differential expression analysis.

Transgenic *Prnp* and *Sprn* FVB/N knockout mouse lines were already described [2, 4, 7]. Wild type (WT) FVB/N mice were purchased from Janvier [8]. ShRNA lentiviral vector solutions were purchased from Sigma with infectious titers over 10^9^ infectious units/mL (LS1: TRCN0000179960 and LS2: TRCN0000184740 against *Sprn* transcripts, LP1: TRCN0000319687 and LP2: TRCN 0000273801 against *Prnp* transcripts). Intra-perivitellin space injections and transplantation into pseudo-pregnant recipient mice were performed as previously described [2]. Around 50 one-cell stage embryos were injected for each genotype and lentiviral solution combination (Figure 1A).

### Embryo collection and transcriptomic analyses

Embryos were collected at E6.5 (Figure 1B). Total RNA was isolated from pools of 6–14 E6.5 embryos, deriving from 3 to 5 females. RNA extractions and integrity analysis were performed as previously described [2]. Three to four independent pools were produced for each experimental group (i.e., genotype and lentiviral solution combination) and analysed using Agilent SurePrint G3 gene expression V2 8 × 60 K mouse microarrays (AMADID: 074809, Figure 1C). All steps were performed by the @BRIDGe facility (INRAE Jouy-en-Josas, France), as described previously [9].

All analyses were performed with R version 4.0.0. Median pixel intensity and local background intensity were read and pre-processed from the raw Agilent files using the R/Bioconductor package *limma* (version 3.44.1, [10, 11]). Probe intensities were quantile-normalized and log_2_-transformed [12]. Using the “gIsWellAboveBackground” flag, non-control probes were called as present if they were above background in at least 3 samples. After averaging intensities for remaining probes with identical target sequences, a single representative probe was chosen for each gene according to the maximum observed variance across samples (Figure 1C).

### Hallmark gene set analyses

To evaluate the potential role played by specific ensembles of gene sets, hallmark gene sets from the Molecular Signatures Database (MSigDB, [13, 14]) were obtained for *Mus musculus* using the *msigdbr* package (version 7.2.1). Among the 50 available gene sets, we focused our attention on a subset of 14 hallmark gene sets related to PrP recognized physiological functions (see below). Comparisons of interest for the hallmark gene set analysis were defined for four different experimental groups as compared to WT mice: (1) *Prnp*^*0/0*^*Sprn*^*0/0*^; (2) *Prnp*^0/0^*Sprn*^*0/0*^*Sprn*^*KD*^; (3) *Prnp*^0/0^*Sprn*^*0/0*^*Prnp*^*KD*^; and (4) *Prnp*^0/0^*Sprn*^*KD*^. To minimize possible off-target effects, contrasts for comparisons with groups (2)-(4) were constructed by averaging over the two lentiviruses for each gene knockdown. Using the *fry* self-contained rotation gene set test from *limma* [15], we sought to identify whether genes in each selected hallmark gene set were globally differentially expressed for a given comparison (Figure 1D). *P*-values were calculated corresponding to tests for gene sets exhibiting significant over-expression (“Up”) and under-expression (“Down”), as well as differential expression regardless of direction (“Mixed”). Raw *P*-values were corrected for multiple testing using the Benjamini–Hochberg approach to control the false discovery rate (FDR, [16]), and gene sets were identified as significantly globally differentially expressed if their adjusted *P-*value < 0.05.

### Differential expression analysis

For the differential analysis (Figure 1E), a linear model with group-means parameterization (i.e., no intercept and a separate coefficient for each group) was fit for each gene. Using *limma*, an empirical Bayes approach was used to moderate the standard errors of the estimated log-fold changes. Contrasts were defined to identify differentially expressed genes for each comparison of interest; we focused in particular on the comparison of *Prnp*^*0/0*^*Sprn*^*0/0*^ and WT E6.5 embryos. As before, *P-*values were corrected for multiple testing using the Benjamini–Hochberg approach to control the false discovery rate [16], and genes were identified as significantly differentially expressed if their adjusted *P*-value < 0.05 and absolute log fold change > 1.

### RT-qPCR analysis

Reverse transcription was performed on 500 ng of total RNA from the 4 pools of WT and the 3 pools of *Prnp*^*0/0*^*Sprn*^*0/0*^ E6.5 embryos previously used for transcriptomic analysis (see the “Embryo collection and transcriptomic analyses” section) using InVitrogen SuperScript™ IV Vilo™ reverse transcriptase kit (11766500) and random primers, according to the manufacturer’s instructions. RT-qPCR quantification was performed on triplicates using the SYBR Green quantitative PCR kit (Applied Biosystems) and standard PCR conditions. Primers were designed on separate exons to produce 100-bp amplicons, with a Tm of 60 °C. Analyses were performed using the Δ(ΔCt) method (Biogazelle QBasePlus software, Biogazelle NV, Ghent, Belgium) and normalizing genes. The GAPDH and UBC genes were used for normalization, using primers 5′-tgacgtgccgcctggagaaa-3′ and 5′-agtgtagcccaagatgcccttcag-3′ for GAPDH and 5′-cgtcgagcccagtgttaccaccaagaagg-3′ and 5′-cccccatcacacccaagaacaagcacaag-3′ for UBC. Three genes were included for RT-qPCR analyses: *Ada*, using primers 5′-tagacactgactaccagatgac-3′ and 5′-tggctattggtattctctgtag-3′; *Cds2*, using primers 5′-tggatcgctttgactgccagt-3′ and 5′-tgttgaagatgtgaagctgctg-3′; and *Spint1*, using primers 5′-aggaacagcagtgtcttgagt-3′ and 5′-atgcagatgcaacgaaatacag-3′.

### Analysis results

Although *Prnp*^*0/0*^*Sprn*^*0/0*^ mice are viable [3, 4], the knockdown of *Sprn* in *Prnp*^*0/0*^ mouse embryos was reported to induce embryonic lethality highlighted by a developmental failure of the trophectoderm-derived compartment noticeable at E7.5 [2]. We reinvestigated this latter observation by transcriptomic analysis of such embryos at E6.5, focusing on a subset of MSigDB including 14 hallmark gene sets related to PrP recognized physiological functions ([1, 17–19], Table 1). Three of those hallmark gene sets were significantly altered in *Sprn*-knockdown, *Prnp*^*0/0*^ E6.5 embryos compared to their WT counterparts (adjusted *P*-value < 0.05): interferon-α and -γ responses and apoptosis, while inflammatory response was significant with an adjusted *P*-value < 0.10 (Table 1).

**Table 1:** **Hallmark gene set analyses at E6.5.**

Hallmark gene set	WT vs P0S0		WT vs P0S0S-		WT vs P0S0P-		WT vs P0S-	
	Direction	FDR	Direction	FDR	Direction	FDR	Direction	FDR
Adipogenesis	Up	0.62552876	Up	0.80753343	Down	0.79673023	Up	0.79147888
**Apoptosis**	Down	0.93132096	Down	0.17156136	Down	0.16934382	**Down**	**0.01640416**
Cholesterol homeostasis	Up	0.90561461	Down	0.85373899	Down	0.49413621	Down	0.71885689
E2F targets	Down	0.90561461	Up	0.19319905	Up	0.31979891	Up	0.11180027
Epithelial mesenchymal transition	Down	0.93132096	Down	0.4305476	Down	0.31979891	Down	0.1308924
Hypoxia	Up	0.90561461	Down	0.42575279	Down	0.18867174	Down	0.11180027
*Inflammatory response*	Up	0.90561461	Down	0.10111071	Down	0.18867174	*Down*	*0.07054624*
**Interferon alpha response**	Down	0.90561461	**Down**	**0.00193216**	**Down**	**0.00379274**	**Down**	**0.00041329**
**Interferon gamma response**	Down	0.90561461	**Down**	**0.00463537**	**Down**	**0.00972725**	**Down**	**0.00075138**
Notch signaling	Down	0.90561461	Up	0.80753343	Up	0.82613941	Down	0.9957742
**Reactive oxygen species pathway**	Up	0.90561461	**Down**	**0.01564074**	Down	0.18867174	Down	0.17771965
TGF beta signaling	Up	0.90561461	Down	0.57494044	Down	0.557864	Down	0.79147888
Wnt beta catenin signaling	Down	0.90561461	Up	0.19319905	Up	0.18867174	Up	0.10479931
Xenobiotic metabolism	Up	0.62552876	Down	0.80753343	Down	0.68538223	Up	0.95587952

Top margin: Compared genotypes. P0: *Prnp*^*0/0*^. SO: *Sprn*^*0/0*^. S-: knockdown of *Sprn*. P-: knockdown of *Prnp*. For each knockdown, two independent lentiviral ShRNA vectors were used (see the “Transgenic lines and lentiviral inoculations” section). Left margin: hallmark gene sets [13, 14]. Significantly altered hallmark gene sets are highlighted in boldface (FDR < 0.05) and italicized (FDR < 0.10)

We similarly investigated the transcriptomic outcomes at E6.5 of *Sprn*- or *Prnp*-knockdown in *Prnp*^*0/0*^*Sprn*^*0/0*^ mouse embryos. The knockdowns of *Sprn* or *Prnp* were performed on a knockout genotype for both of these genes to highlight only those pathways associated with lentiviral ShRNA vector infections on this specific genetic background. Two different ShRNAs were again used for each targeted gene to reduce the likelihood of observing an off-target-induced biological disturbance. Compared to WT E6.5 embryos, only two hallmark gene sets were consistently and significantly altered: interferon-α and -γ responses (Table 1). However, compared to the previous analysis, the statistical significance of these gene sets was unexpectedly reduced by tenfold. Furthermore, no apoptosis induction was detected (Table 1).

We next compared the transcriptome of E6.5 WT and *Prnp*^*0/0*^*Sprn*^*0/0*^ mouse embryos. Only 11 genes were found to be differentially expressed between these two genotypes with an adjusted *P*-value < 0.05 and absolute log fold change > 1 (Table 2). All 11 of these genes were similarly found to be significantly differentially expressed in the same direction in *Sprn*-knockdown, *Prnp*^*0/0*^ compared to WT E6.5 mouse embryos, albeit with weaker log fold changes for the majority. As already discussed, the *Prnp* and *Sprn* gene invalidations did not induce alteration of their transcript expression levels, and their absence in this list was thus expected [4, 7]. Most of the differentially expressed genes were reported to be transcribed in the embryo ectoderm and mesenchyme, and only a few in the endoderm or in the extraembryonic component (Table 2, [20]).

**Table 2:** **Differentially expressed genes between *Prnp***^***0/0***^***Sprn***^***0/0***^** and WT E6.5 mouse embryos.**

Gene name	Description	NCBI gene	Log fold change	Adjusted *P*-value	Embryo ectoderm	Embryo endoderm	Embryo mesenchyme	Extraembryonic component	RT-qPCR *Prnp*^*0/0*^* Sprn*^*0/0*^ E6.5	RT-qPCR WT E6.5	RT-qPCR *P* value
Spint1	Serine protease inhibitor, Kunitz type 1	20732	1.599051	1.96414E−08	✓	✓	✓	✓	29.56 ± 2.1	25.88 ± 4.5	0.126
Gm30906	Long non-coding RNA	102632964	− 1.477668	1.96414E−08	✓		✓				
Scg5	Secretogranin V, secreted chaperone protein	20394	−1.334427	4.2595E−08	✓		✓				
Spg11	Spatacsin vesicle trafficking associated	214585	−1.081024	2.05026E−06	✓		✓				
Cds2	CDP-diacylglycerol synthase 2	110911	1.297156	2.42507E−06	✓		✓	✓	50.87 ± 5.5	26.31 ± 4.8	0.001
Jmjd7	Jumonji domain containing 7	433466	−1.479236	1.31005E−05	✓		✓				
Ada	Adenosine deaminase	11486	−2.117557	4.32324E−05	✓		✓	✓	10.53 ± 1.1	16.08 ± 4.3	0.043
AK148702		RIKEN clone 7120437D13 (MGI:3537747)	2.819679	0.004937838	–	–	–	–			
Cplx2	Complexin 2	12890	2.616736	0.010457547	✓		✓				
Gm10734		RIKEN clone I530011G18 (MGI:3565867)	−1.104109	0.010457547	✓		✓				
Sdc4	Syndecan 4	20971	−1.157117	0.021156447	✓		✓				

Results are shown for significantly differentially expressed genes (FDR < 0.05, absolute log fold change ≥ 1). Checkmarks for each gene represent reported expression in embryo ectoderm, embryo endoderm, embryo mesenchyme, and extraembryonic component [20]. Blank spaces and dashes represent unreported expression and no available data, respectively. Normalized RT-qPCR expression levels are reported in each group for the three tested genes (mean ± standard errors) with the associated *P*-value from a two-sample Student’s *t*-test.

**Figure 2:**
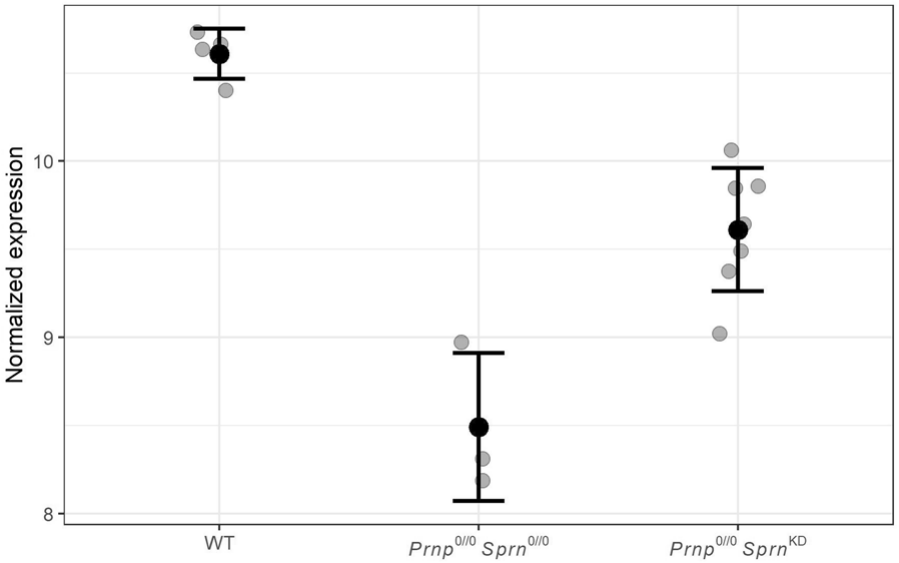
**Normalized expression of *Ada* in WT; *Prnp***^***0/0***^***Sprn***^***0/0***^**; and *Prnp***^***0/0***^***Sprn***^***KD***^** mice.** Values are represented as dot plots for individual samples (grey points) with means (black points) and standard deviations (bars) for each experimental group.

Finally, we focused our attention on the three genes expressed in the extraembryonic component (*Spint1*, *Cds2*, *Ada*); in particular, *Ada* exhibited strong differential expression (log fold-change = −2.1, Table 2, Figure 2). Differential expression of these three genes was further assessed by RT-qPCR analysis. The results validated those obtained from the microarray data for *Ada* and *Cds2*; a similar but insignificant trend was observed for *Spint1* (Table 2).

## Discussion

Our results confirmed that knockdowns of *Sprn* in *Prnp*^*0/0*^ mouse embryos induce apoptosis alongside interferon responses at E6.5, in accord with previous reports and suggesting that embryonic lethality could be diagnosed at earlier developmental stages. Because two different ShRNAs were used, targeting different regions of the *Sprn* transcript, it is unlikely that apoptosis results from an off-target effect.

A natural subsequent question is whether this apoptotic induction, alongside inflammatory and interferon responses, could result from the association of a lentiviral ShRNA-expressing vector inoculation [21] with a *Prnp*-knockout induced interferon-primed state [22] in the absence/reduction of *Sprn* expression, which has been shown to be required to induce apoptosis [2]. In our study, we found that *Prnp* or *Sprn* knockdown in mouse *Prnp*^*0/0*^*Sprn*^*0/0*^ embryos induces reduced interferon responses and no apoptosis at E6.5*.* These results could suggest that the expression or the knockout of *Sprn* is required to avoid lentiviral ShRNA vector induction of a strong interferon response associated with apoptosis in *Prnp*^*0/0*^ mouse embryos, while its knockdown exacerbates these pathways. A potential explanation for these apparent contradictory observations is that the knockout of the two genes induces a genetic adaptation that in turn helps control the lentiviral-induced responses. Such an adaptation might not take place with the *Sprn*-knockdown or to an insufficient level.

To assess this hypothesis, we compared the gene expression of WT and *Prnp*^*0*/0^*Sprn*^*0*/0^ E6.5 embryos**,** revealing highly similar transcriptomes with only 11 differentially expressed genes. Since adult expression of both *Prnp* and *Sprn* genes is more abundant in the nervous system, and since PrP involvement in muscle and bone development/regeneration has been previously reported, deregulation of these genes in the ectoderm and in the mesenchyme might be relevant observations. However, in *Sprn*-knockdown, *Prnp*^*0/0*^ embryos, a developmental failure of the trophectoderm-derived compartment was reported [2], instead suggesting a major role of the extraembryonic component in the appearance of this lethality. Only 3 out of the 11 differentially expressed genes (*Spint1*, *Cds2*, *Ada*) are known to be expressed in the extraembryonic component. *Spint1* was recently reported to be a biomarker of placental insufficiency [23]. Low circulating levels of Spint1 are associated with placental failure whereas here, at E6.5, this expression is higher in *Prnp*^*0/0*^*Sprn*^*0/0*^ embryos compared to their WT counterparts. Whether *Spint1* overexpression can favor placental development remains to be demonstrated. *Cds2* is a widely expressed gene indirectly involved in the positive control of angiogenesis [24]. Its overexpression in *Prnp*^*0/0*^*Sprn*^*0/0*^ embryos could suggest a sustained angiogenesis of the placenta, but in the absence of associated deregulation of co-factors, such as vascular endothelial growth factors, the interpretation of this observation remains fragile. Nevertheless, the differential expression of the two above-mentioned genes appears to favor placental development and to contribute to the survival of the *Prnp*^*0/0*^*Sprn*^*0/0*^ mouse embryos. However, their potential implication in the control of the interferon response remains elusive.

The third strongly differentially expressed gene transcribed in the extraembryonic component was *Ada*. Disruption of the *Ada* gene in mice induces perinatal lethality [25], a phenotype rescued by tissue-specific placental expression of this gene [26]. Its crucial role in the trophectoderm-derived compartment was also indirectly emphasized through the knockout of the AP-2γ transcription factor-encoding gene that resulted in an early embryonic lethal phenotype, similar to that observed for *Sprn*-knockdown in *Prnp*^*0/0*^ embryos [2, 27], associated with a lack of *Ada* gene expression in the extraembryonic cells [28]. However, in *Prnp*^*0/0*^*Sprn*^*0/0*^ mouse embryos, only a downregulation of the *Ada* gene expression is observed, thus likely avoiding the occurrence of these drastic phenotypes. It should be mentioned that *Ada*^*0/*+^ mouse embryos were similarly not reported to be affected [25]. Interestingly, Ada congenital defect induces a severe combined immunodeficiency syndrome [27]. Expression levels of this enzyme correlate with the production levels of interferons and proinflammatory factors, and modulation of Ada activity was even proposed as a potential therapeutic target [28–31]. High interferon responses can induce side effects among which some, such as autoimmune reactions, can be detrimental. The control of the interferon response is thus crucial, and as already mentioned, altered in the absence of members of the prion protein family [17]. The downregulation of the *Ada* gene expression observed in *Prnp*^*0/0*^*Sprn*^*0/0*^ mouse embryos might help to control the interferon and inflammatory responses induced by lentiviral ShRNA-encoding vector infections to a level compatible with their survival. This genetic adaptation is only partially induced in *Sprn*-knockdown, *Prnp*^*0/0*^ embryos, resulting in a high rate of embryonic lethality [2].

An alternative explanation would be that expression of a ShRNA targeting the invalidated gene in knockout mice does not induce specific immune response and antiviral functions due to the absence of knockdown-induced RNA degradation products [32]. However, the design of the *Prnp* and *Sprn* gene invalidations was such that their transcription remains unaffected, while the produced mRNA, which still encompasses the ShRNA target site, no longer encodes for the protein. As RNA expression was confirmed at embryonic stages for *Prnp* [33] and *Sprn* [7] knockout mice, this hypothesis is thus unlikely.

Overall, our results suggest a genetic adaptation of *Prnp*^*0/0*^*Sprn*^*0/0*^ mouse embryos, both to sustain placental physiology that is affected in the absence of PrP [31] or Shadoo [7] and to refrain the upregulation of induced interferon responses following environmental stresses. This genetic adaptation might involve the downregulation of *Ada* and its related pathways, this protein being involved in immunomodulation and ectoplacental development. Although this hypothesis remains to be further supported by direct experiments, it offers an explanation for the discrepancy observed between knockdowns and knockouts in previously reported data [2, 3] and adds to the list of knockout genotypes that have acquired genetic adaptation.

## Data Availability

Raw microarray data files and all analysis scripts needed to pre-process the data and reproduce the analyses described in this work are openly available on the Data INRAE portal [34].
